# Induction of cellulase production by Sr^2+^ in *Trichoderma reesei* via calcium signaling transduction

**DOI:** 10.1186/s40643-022-00587-3

**Published:** 2022-09-06

**Authors:** Ni Li, Yi Zeng, Yumeng Chen, Yaling Shen, Wei Wang

**Affiliations:** The State Key Laboratory of Bioreactor Engineering, New World Institute of Biotechnology, East China University of Science and Technology, Shanghai, 200237 China

**Keywords:** *Trichoderma reesei*, Sr^2+^, Cellulase, ROS, Calcium signaling, Signal transduction

## Abstract

*Trichoderma reesei* RUT-C30 is a well-known high-yielding cellulase-producing fungal strain that converts lignocellulose into cellulosic sugar for resource regeneration. Calcium is a ubiquitous secondary messenger that regulates growth and cellulase production in *T. reesei*. We serendipitously found that adding Sr^2+^ to the medium significantly increased cellulase activity in the *T. reesei* RUT-C30 strain and upregulated the expression of cellulase-related genes. Further studies showed that Sr^2+^ supplementation increased the cytosolic calcium concentration and activated the calcium-responsive signal transduction pathway of Ca^2+^–calcineurin-responsive zinc finger transcription factor 1 (CRZ1). Using the plasma membrane Ca^2+^ channel blocker, LaCl_3_, we demonstrated that Sr^2+^ induces cellulase production via the calcium signaling pathway. Supplementation with the corresponding concentrations of Sr^2+^ also inhibited colony growth. Sr^2+^ supplementation led to an increase in intracellular reactive oxygen species (ROS) and upregulated the transcriptional levels of intracellular superoxide dismutase (s*od1*) and catalase (*cat1*). We further demonstrated that ROS content was detrimental to cellulase production, which was alleviated by the ROS scavenger *N*-acetyl cysteine (NAC). This study demonstrated for the first time that Sr^2+^ supplementation stimulates cellulase production and upregulates cellulase genes via the calcium signaling transduction pathway. Sr^2+^ leads to an increase in intracellular ROS, which is detrimental to cellulase production and can be alleviated by the ROS scavenger NAC. Our results provide insights into the mechanistic study of cellulase synthesis and the discovery of novel inducers of cellulase.

**Graphical Abstract:**

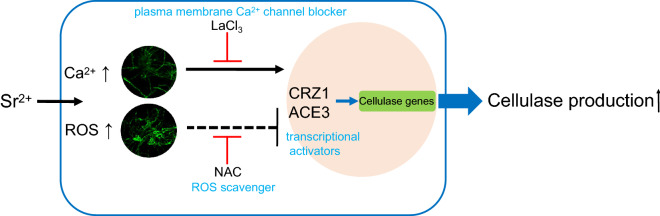

**Supplementary Information:**

The online version contains supplementary material available at 10.1186/s40643-022-00587-3.

## Introduction

Lignocellulose is a well-known renewable biomass energy source that can be degraded to bioethanol (Baldrian and Valášková 2008; Somerville et al. 2010). The economic and practical degradation of cellulose has been widely recognized and accepted (Li et al. 2018). Cellulose degradation is dependent on *Trichoderma reesei*, a strain that efficiently produces cellulase and hemicellulase (Fischer et al. 2021; Li et al. 2016, 2021; Martzy et al. 2021; Yan et al. 2021). *Trichoderma reesei* can produce large amounts of cellulases and hemicellulases using inducible carbon sources (such as Avicel). In contrast, negligible amounts of cellulases and hemicellulases are produced using fast-utilizing carbon sources (such as glucose) (Antoniêto et al. 2016). The production of cellulases is an extremely complex process regulated by many factors, such as transcription factors (Xin et al. 2010) and the second messenger Ca^2+^ (Chen et al. 2019).

Cellulase synthesis in *T. reesei* is regulated by several transcription factors (Zhang et al. 2018). Among these factors, xylanase regulator 1 (XYR1) is involved in activation of the expression of cellulases and hemicellulases (Carle-Urioste et al. 1997; Furukawa et al. 2009; Zeilinger et al. 1996), as well as the regulation of d-xylose metabolism (Stricker et al. 2006), which is considered as a key transactivator (Cao et al. 2019). Deletion of *xyr1* results in a substantial reduction in cellulase and hemicellulase production (Stricker et al. 2006). ACE3, another crucial transcriptional activator, is essential for cellulase and xylanase production (Häkkinen et al. 2014). Deletion of *ace3* is fatal for cellulase gene expression, whereas this deletion slightly reduces the expression of hemicellulases (Luo et al. 2020) and xylanase (Zhang et al. 2019).

Ca^2+^ signaling is a well-known secondary signaling pathway that broadly regulates primary and secondary metabolism in microorganisms (Roy et al. 2021). The intracellular calcium signaling components include free Ca^2+^, calmodulin (CaM), calcineurin (CnA), and calcineurin-responsive zinc finger transcription factor 1 (Crz1) (Bootman et al. 2002). Increases in cytosolic Ca^2+^ levels lead to the activation of Crz1 via Ca^2+^/CaM/CnA, which activates downstream genes with different functions by binding to their promoter regions (Martins-Santana et al. 2020). Chen et al. (2016) reported that the cytosolic Ca^2+^ concentration activates the binding of the transcription factor Crz1 to the cellulase gene *cbh1* and main transcriptional activator of cellulase genes *xyr1*, thereby upregulating cellulase gene expression. Notably, Chen et al. (2018) demonstrated that Mn^2+^ induces a cytosolic Ca^2+^ burst to enhance the expression of cellulase genes through the Mn^2+^/Ca^2+^ exchanger in *T. reesei*. In *Penicillium brevicompactum*, addition of Ca^2+^ increases the production of the secondary metabolite mycophenolic acid (Chen et al. 2021a).

Reactive oxygen species (ROS) are chemically reactive chemicals that contain oxygen and are considered as harmful by-products of aerobic metabolism (Ren et al. 2017). ROS play a critical role in the development of many filamentous fungi, including *Aspergillus nidulans* (Mendoza-Martínez et al. 2017), *Podospora anserina* (Malagnac et al. 2004), and *Neurospora crassa* (Takemoto et al. 2007). Chen et al. (2022) demonstrated that a higher concentration of Mn^2+^ increased ROS production, ultimately leading to increased mycophenolic acid production in *P. brevicompactum*. Liu et al. (2018) observed that the ROS producer H_2_O_2_ and ROS scavenger *N*-acetyl cysteine (NAC) altered intracellular ROS levels in *Ganoderma lucidum*, which is an efficient approach for enhancing the production of ganoderic acid.

Ca^2+^ can promote the transcription of cellulase genes in the hypercellulolytic *T. reesei* strain RUT-C30. Therefore, we investigated whether other ions of group IIA metals can also increase cellulase production. High Mg^2+^ concentrations significantly inhibit growth (Groisman et al. 2013), which was not considered in this study. Furthermore, a previous study on Ca^2+^ was conducted in *T. reesei* RUT-C30. Therefore, in the current study, the effect of Sr^2+^ on cellulase production was investigated using RUT-C30*.* The effects of Sr^2+^ on mycelial growth and cellulase production were measured, and the transcript levels of *crz1* and intracellular Ca^2+^ levels were evaluated to determine the role of calcium signaling in response to Sr^2+^. This study provides new perspectives for improving cellulase production and insights into the mechanism of cellulase regulation in *T. reesei*.

## Materials and methods

### Strains and growth conditions

*Trichoderma reesei* RUT-C30 (ATCC 56765) and QM6a (ATCC 13631) were used for the experiments. All *T. reesei* strains were cultured on potato dextrose agar (PDA) plates at 28 °C in the dark. Fresh conidia were washed with PDA plates and inoculated. MA medium (Zhang et al. 2019) was used for the general fungal culture. Minimal medium (MM; (NH_4_)_2_SO_4_ 5 g/L; KH_2_PO_4_ 15 g/L; urea 0.3 g/L; MgSO_4_ 0.6 g/L; CaCl_2_ 0.6 g/L; FeSO_4_·7H_2_O 5 mg/L; CoCl_2_·6H_2_O 2 mg/L; MnSO_4_·H_2_O 1.6 mg/L; ZnSO_4_·7H_2_O 1.4 mg/L; pH 5.5) (Chen et al. 2018) with 2% (w/v) glucose or 1% (w/v) Avicel was used to assess the effect of Sr^2+^ on hyphal growth and cellulase production. Transfer experiments were performed to study the effects of Sr^2+^ on cellulase production. The conidia were first cultured in 100 mL MA medium containing 2% glucose at 28 °C for 36 h to form mycelia. Mycelia (1 mL) were collected by centrifugation, washed with MM without a carbon source, and transferred into 50 mL of fresh MM containing 1% (w/v) Avicel (PH-101; Sigma-Aldrich, St. Louis, MO, USA) with the addition of SrCl_2_ to final concentrations of 0–120 mM.

### Enzyme activity analysis and fungal growth

The culture (1 mL) was collected and centrifuged at 14,000×*g* at 4 °C for 5 min. The resulting supernatant was used to determine cellulase activity. *p*NPCase, CMCase, FPase, and xylanase activities were measured as previously reported (Zhang et al. 2016; Liu et al. 2019). The Detergent Compatible Bradford Protein Assay Kit (Beyotime, Shanghai, China) was used to determine protein concentrations. Biomass was measured indirectly using the intracellular protein method (Bischof et al. 2013) with some modifications. Three parallel samples (1 mL each) was collected, and the mycelia were collected by filtration, lysed with 1 M NaOH for 2 h, and centrifuged to obtain the intracellular proteins. The BCA Protein Concentration Assay Kit (Beyotime, Shanghai, China) was used to determine the total protein concentration. The biomass dry weight was calculated as 0.32 g intracellular protein per gram of dry cell weight (Chen et al. 2018).

For fungal growth experiments, fresh conidia were diluted to 2.5 × 10^6^/mL with sterile water, inoculated an equal volume of conidia fluid (2 μL) into the center of MM plates, and incubated at 28 °C in the dark for 4 days.

### RNA extraction and RT‑qPCR

RNA isolation and reverse transcription quantitative polymerase chain reaction (RT‑qPCR) analyses were performed as described by Zhang et al. (2018) with some modifications. Briefly, total RNA from mycelia was extracted using the FastRNA Pro Red Kit (MPbio, Irvine, CA, USA) according to the manufacturer’s instructions. cDNA was synthesized from total RNA using the TransScript Uni All-in-One First-Strand cDNA Synthesis SuperMix for qPCR (TransGen Biotech, Beijing, China). For RT-qPCR, the transcriptional levels of *cbh1* (encoding cellobiohydrolase I), *egl1* (encoding endoglucanase I), *xyr1* (encoding the main factor XYR1), *ace3* (encoding the main factor ACE3), *crz1* (calcineurin-responsive zinc finger transcription factor 1, Trire2:36391), *sod1* (copper/zinc superoxide dismutase, Trire2:123029), and *cat1* (catalase, Trire2:70600) were analyzed using PerfectStart™ Green qPCR SuperMix (TransGen Biotech). The 2^−ΔΔCt^ method was used for calculations (Livak and Schmittgen 2001). The *sar1* gene was used as an internal reference to normalize the data. The primers used for RT-qPCR are described in Additional file 2: Table S1.

### Labeling and detection of cytosolic Ca^2+^ and ROS

Cytosolic Ca^2+^ and ROS levels were assessed according to the manufacturer’s instructions, with some modifications. For fluorescent detection of Ca^2+^ and ROS, the mycelia were incubated with the fluorescent probe Fluo-4 AM/DCHF-DA (Beyotime, Shanghai, China) at 28 °C for 30 min. The mycelia were washed three times with phosphate-buffered saline (PBS) (pH 5.0) to remove excess fluorophores and avoid excessive background noise. Images of the DCF- and Fluo-4 AM-labeled mycelia were visualized using an S Plan Fluor ELWD 20×, 0.5 numerical aperture (NA) objective and a digital sight camera on an Eclipse Ti inverted microscope system (Ti-E; Nikon, Tokyo, Japan), comprising a FITC filter (420–490 nm band-pass excitation filter and an emission filter of 535 nm). The average fluorescence intensities were analyzed using ImageJ software (National Institutes of Health, Bethesda, MD, USA).

### Chemical treatments

To evaluate the effect of Sr^2+^ on cellulase production, different concentrations of SrCl_2_ were added immediately after transferring the mycelia to fresh MM. To evaluate the roles of cytosolic Ca^2+^ and ROS under Sr^2+^ stress, the mycelia were treated with the ROS scavenger NAC, hydrogen peroxide (H_2_O_2_), and plasma membrane Ca^2+^ channel blocker (LaCl_3_). NAC was added immediately after the mycelia were transferred to fresh MM. LaCl_3_ and H_2_O_2_ were added at 24 h after the mycelia were transferred to fresh MM.

### Statistical analysis

All experimental data were obtained with at least three parallel samples with similar or identical results. Error values represent the standard deviation (SD) from the mean of three replicates. Student’s *t*-test was used to compare two samples to detect statistical significance. Duncan’s multiple-range test was used for multiple comparisons. Differences were considered as significant at *p* < 0.05.

## Results

### Effects of Sr^2+^ on *T. reesei* growth

**Fig. 1:**
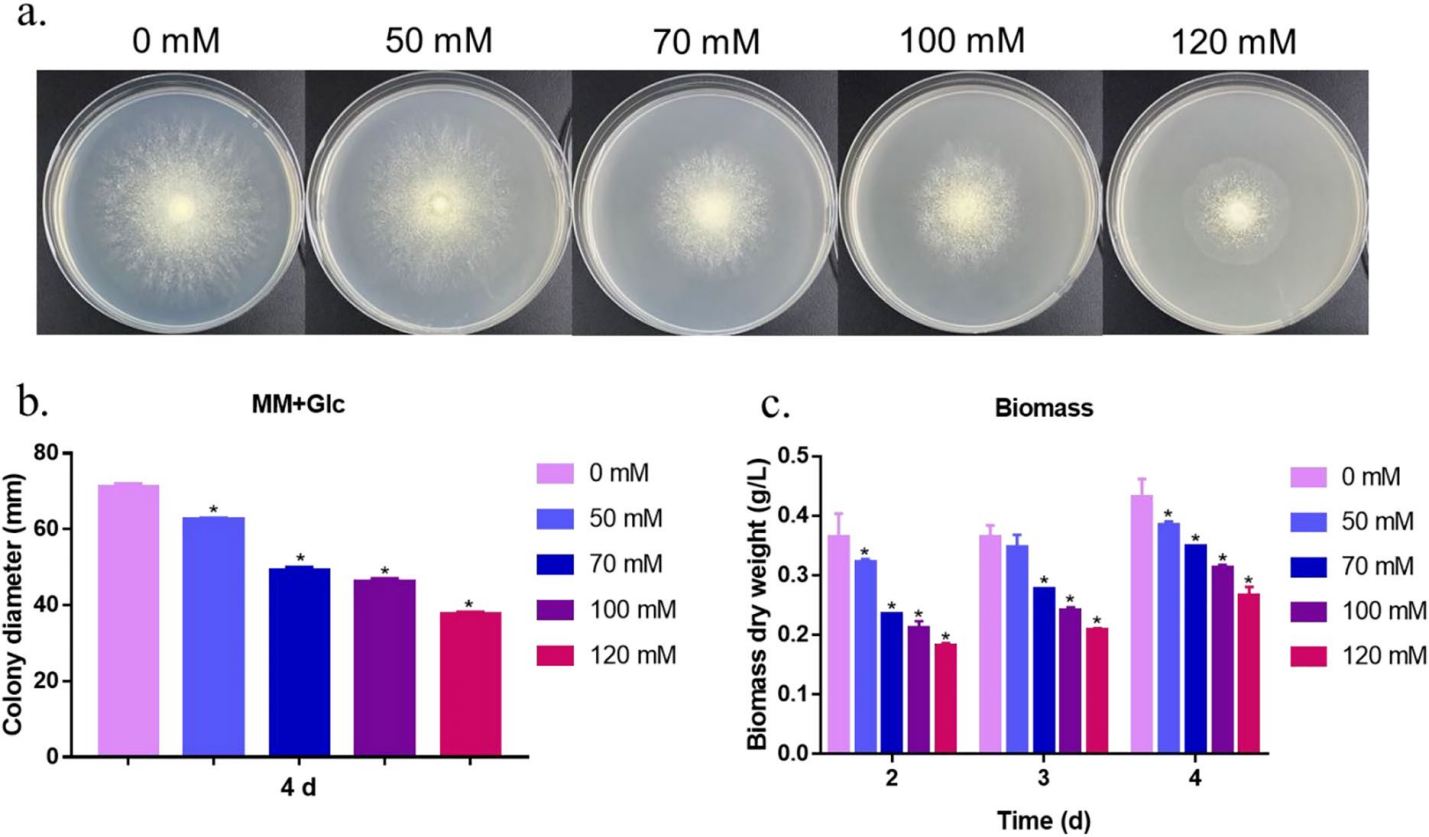
Effect of different concentrations of Sr^2+^ on hyphal growth in *T. reesei* RUT-C30 strain. **a** Hyphal growth of RUT-C30 strain on MM plates. The MM culture was supplemented with Sr^2+^ at a final concentration of 0–120 mM. **b** Colony diameter under different concentrations of Sr^2+^. **c** Biomass dry weight in MM liquid culture of RUT-C30 strain. The final values are presented as the mean ± standard deviation (SD) of three independent experimental results. Asterisks indicate significant differences compared to the control (*p* < 0.05, according to Student’s *t-*test). MM, minimal medium

To investigate the effect of Sr^2+^ on colony growth, different concentrations of Sr^2+^ (0–120 mM) were added to solid MM containing 20 g/L glucose as the sole carbon source. An equal amount of fresh RUT-C30 conidia was spotted onto poured plates. The growth status of mycelia after Sr^2+^ supplementation is shown in Fig. 1a. The *T. reesei* strains grew more slowly as the Sr^2+^ concentration was increased. As shown in Fig. 1b, hyphal growth significantly differed between the 50 mM Sr^2+^ and no Sr^2+^ supplementation groups. Treatment with 70 and 100 mM Sr^2+^ caused a 31% and 35.2% reduction in colony diameter, respectively, compared to that of untreated strains. When the Sr^2+^ concentration was increased to 120 mM, the treated strains exhibited the most severe reduction in colony diameter (approximately 46.9%). This result was consistent with the biomass production data for MM liquid culture with 1% (w/v) Avicel as the sole carbon source (Fig. 1c). These results indicate that supplementation with large amounts of Sr^2+^ inhibited the growth of *T. reesei* strains under both glucose and Avicel carbon sources.

### Cellulase production increased under Sr^2+^ pressure in *T. reesei*

**Fig. 2:**
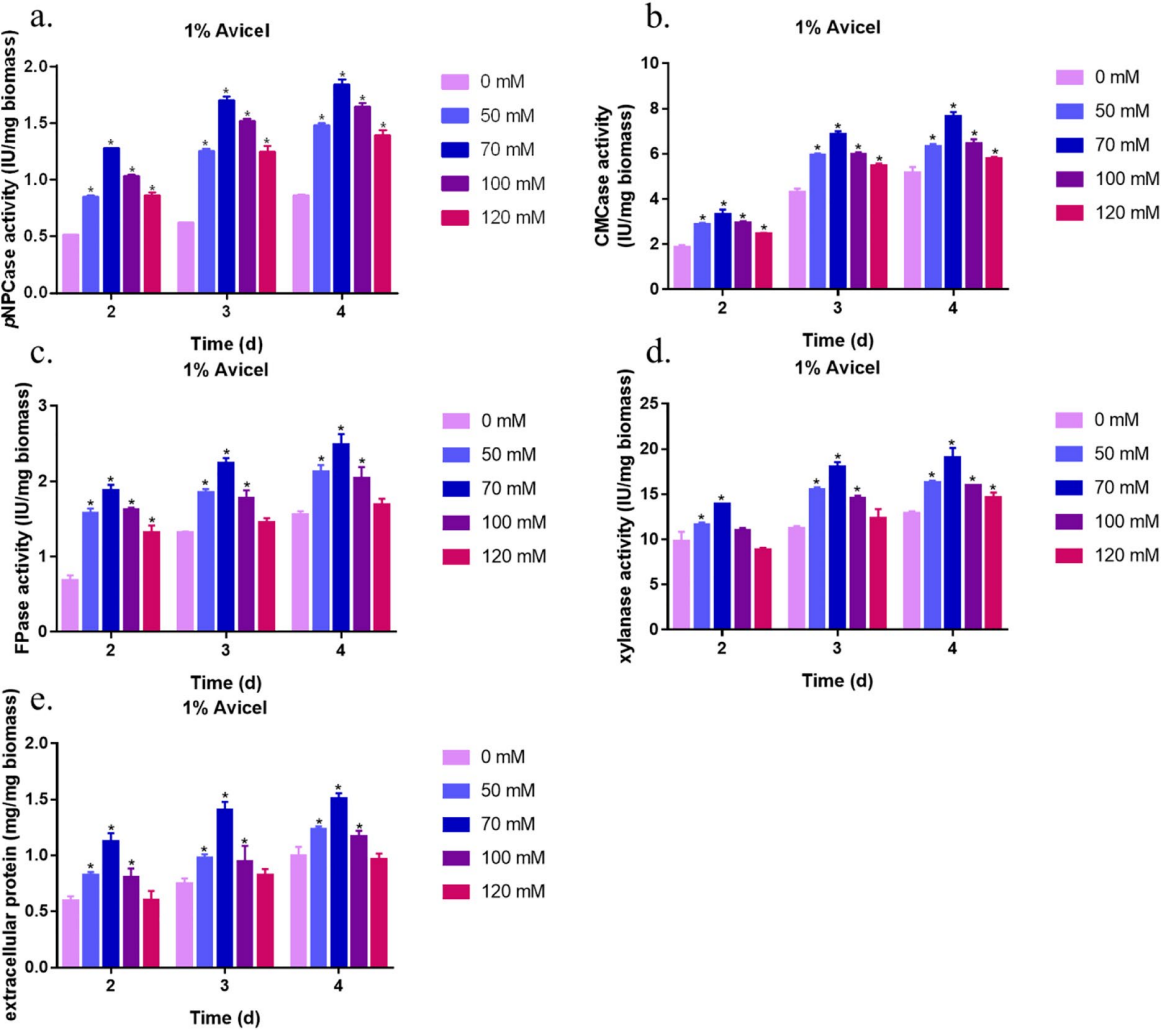
Effect of Sr^2+^ on cellulase production in RUT-C30 strain. *p*NPCase activity (**a**), CMCase activity (**b**), FPase activity (**c**), xylanase activity (**d**), and total protein concentrations (**e**) of the RUT-C30 strain were examined after culture in MM for 2, 3, or 4 days containing different concentrations of Sr^2+^ (0–120 mM). The final values are presented as the mean ± standard deviation (SD) of three independent experimental results. Asterisks indicate significant differences compared to the control (*p* < 0.05, according to Student’s *t-*test). MM, minimal medium

To evaluate the effect of Sr^2+^ on cellulase production, the same amount of RUT-C30 mycelia was transferred into liquid MM with 1% (w/v) Avicel as the carbon source and different concentrations of Sr^2+^ (0–120 mM). As shown in Fig. 2a–e, addition of different concentrations of Sr^2+^ significantly improved cellulase activity and total protein secretion. Addition of 70 mM Sr^2+^ resulted in the largest improvement and significantly increased the *p*NPCase, CMCase, FPase, and xylanase activities, as well as the total protein concentration by 176%, 59.6%, 69.6%, 60%, and 87.2%, respectively, compared to that in the control (without Sr^2+^) on day 3 of cultivation. These results indicate that addition of 50–120 mM Sr^2+^ significantly improved the cellulase yield. The optimal concentration of Sr^2+^ for enhancing cellulase production was 70 mM in RUT-C30, which was used in subsequent analyses.

**Fig. 3:**
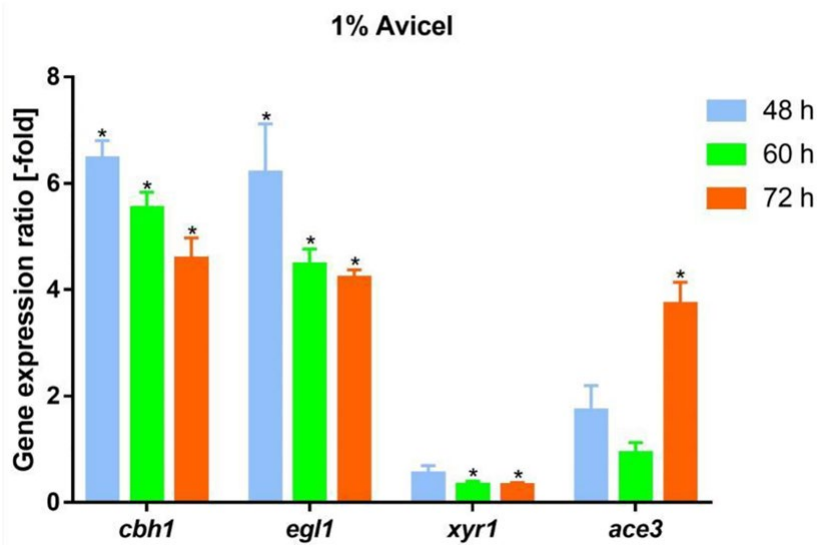
Effect of Sr^2+^ on cellulase-related gene transcription levels in RUT-C30 strain. Gene expression ratios [-fold] of *cbh1*, *egl1*, *xyr1*, and *ace3* in RUT-C30 treated with 70 mM Sr^2+^ relative to RUT-C30 without treatment on 1% Avicel for 48, 60, or 72 h. Gene expression ratios [-fold] were normalized to the corresponding gene expression at the same timepoint in the control (without Sr^2+^). The final values are represented as the mean ± standard deviation (SD) of three independent experimental results. Asterisks indicate significant differences, representing gene expression ratio greater than twofold or less than 0.5-fold between the treated samples and those without Sr^2+^ treatment

To further investigate the effect of Sr^2+^ on cellulase synthesis, the expression levels of two vital cellulase genes (*cbh1* and *egl1*) and essential cellulase transcription activators *xyr1* and *ace3* were detected using RT-qPCR, as shown in Fig. 3. The transcription levels of *cbh1* and *egl1* increased by 546% and 520%, respectively, at 48 h, which was consistent with the upregulation of enzyme activities after addition of 70 mM Sr^2+^ (Fig. 2a–e). Addition of Sr^2+^ upregulated the expression of cellulase activator *ace3* by approximately 72.3% at 48 h (Fig. 3). At 72 h, the expression level of *ace3* increased to 272% compared to that in the control. These results indicate that Sr^2+^ supplementation significantly increased the transcription levels of cellulase genes and the essential cellulase transcription activator *ace3*. However, addition of Sr^2+^ led to downregulation of *xyr1* expression (approximately 46%) at 48 h (Fig. 3).

### Cytosolic Ca^2+^ accumulation in *T. reesei* was induced by high Sr^2+^ concentrations

**Fig. 4:**
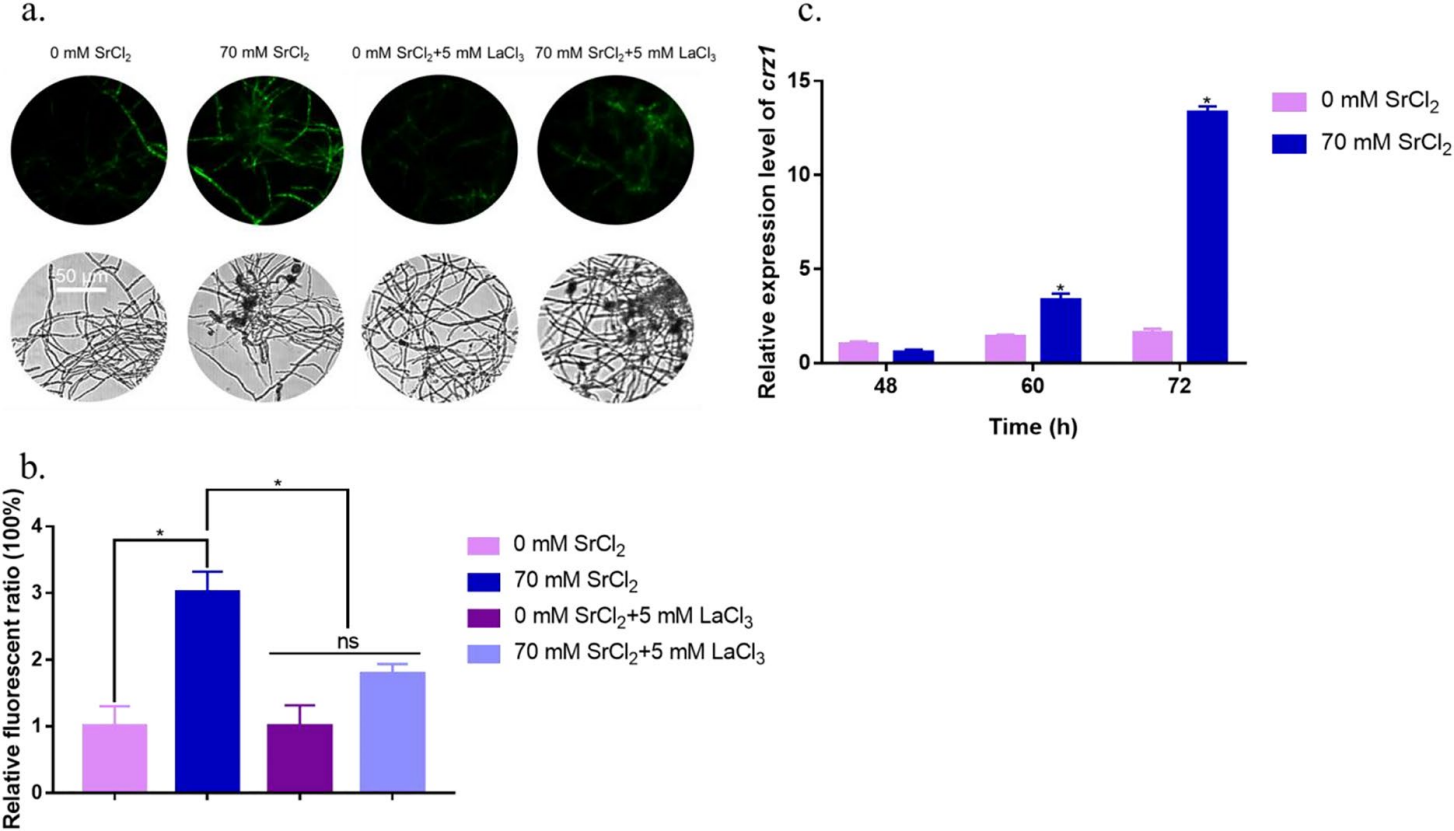
Cytosolic Ca^2+^ levels increase after Sr^2+^ addition. **a** Cytosolic Ca^2+^ levels were detected using the specific fluorescent probe Fluo-4 AM. *Trichoderma reesei* RUT-C30 strain was cultured in MM for 2 days with or without supplementation of the Ca^2+^ channel inhibitor LaCl_3_ and 0 or 70 mM Sr^2+^. To treat hyphae, 4 μM Fluo-4 AM was used. For detection, automatic inverted fluorescence microscopy was used to monitor the fluorescence intensity. Stronger green fluorescence indicated a higher intracellular Ca^2+^ content. **b** Comparative fluorescence ratios demonstrating the effects of LaCl_3_ on the cytosolic Ca^2+^ burst induced by Sr^2+^. The *y*-axis represents the Ca^2+^ fluorescence ratio measured by CLSM, and the *x*-axis represents different treatments with Sr^2+^ and LaCl_3_. **c** Transcriptional levels of *crz1* after treatment with 0 or 70 mM Sr^2+^ for 48, 60, or 72 h were also detected. The final values are presented as the mean ± standard deviation (SD) from three independent experimental results. Asterisks indicate significant differences compared to the strain without Sr^2+^ treatment (*p* < 0.05, according to Student’s *t-*test). DIC, differential interference contrast; MM, minimal medium

Cytosolic concentrations of Ca^2+^ were detected using the fluorescent probe Fluo-4 AM, which emits green fluorescence after crossing the cell membrane and binding to cytosolic Ca^2+^ (Chen et al. 2021b). The fluorescence intensity represents the relative amount of free intracellular Ca^2+^. As shown in Fig. 4a, following addition of 70 mM Sr^2+^, the green fluorescence intensity in RUT-C30 cells was stronger than that in the control (without Sr^2+^ treatment). Analysis using ImageJ software showed that the fluorescence intensity of the experimental group increased by twofold after addition of Sr^2+^ (Fig. 4b), suggesting that the cytosolic Ca^2+^ content increased after Sr^2+^ treatment.

Increased concentrations of the second messenger Ca^2+^ in the cytosol lead to a response in the downstream calcium pathway (Li et al. 2019; Liu et al. 2019). Crz1, a critical activator of the calcium signal transduction terminal, plays an essential role in calcium signal transduction pathways (Chen et al. 2016; Martins-Santana et al. 2020). The transcription level of the transcriptional regulator *crz1* detected using RT-qPCR was upregulated after Sr^2+^ induction (Fig. 4c).

A plasma membrane Ca^2+^ channel blocker, LaCl_3_ (Zhang et al. 2022), which prevents the influx of extracellular calcium ions, was used to examine the effect of Sr^2+^ on the cytosolic Ca^2+^ burst. As illustrated in Fig. 4a, b, the significant increase in intracellular Ca^2+^ induced by Sr^2+^ was markedly weakened after supplementation with LaCl_3_. At 5 mM LaCl_3_, the fluorescence intensity of the mycelia decreased by 40.6% compared with that in the absence of LaCl_3_. However, LaCl_3_ supplementation only negligibly reduced the fluorescence intensity compared with that in the control without Sr^2+^ (Fig. 4b).

These results indicate that Sr^2+^ induces a cytosolic calcium burst and results in calcium signal transduction, and that adding a plasma membrane Ca^2+^ channel blocker can block the Sr^2+^-induced calcium burst (Fig. 4b).

### Sr^2+^ induced cytosolic Ca^2+^ burst and substantially increased cellulase production

**Fig. 5:**
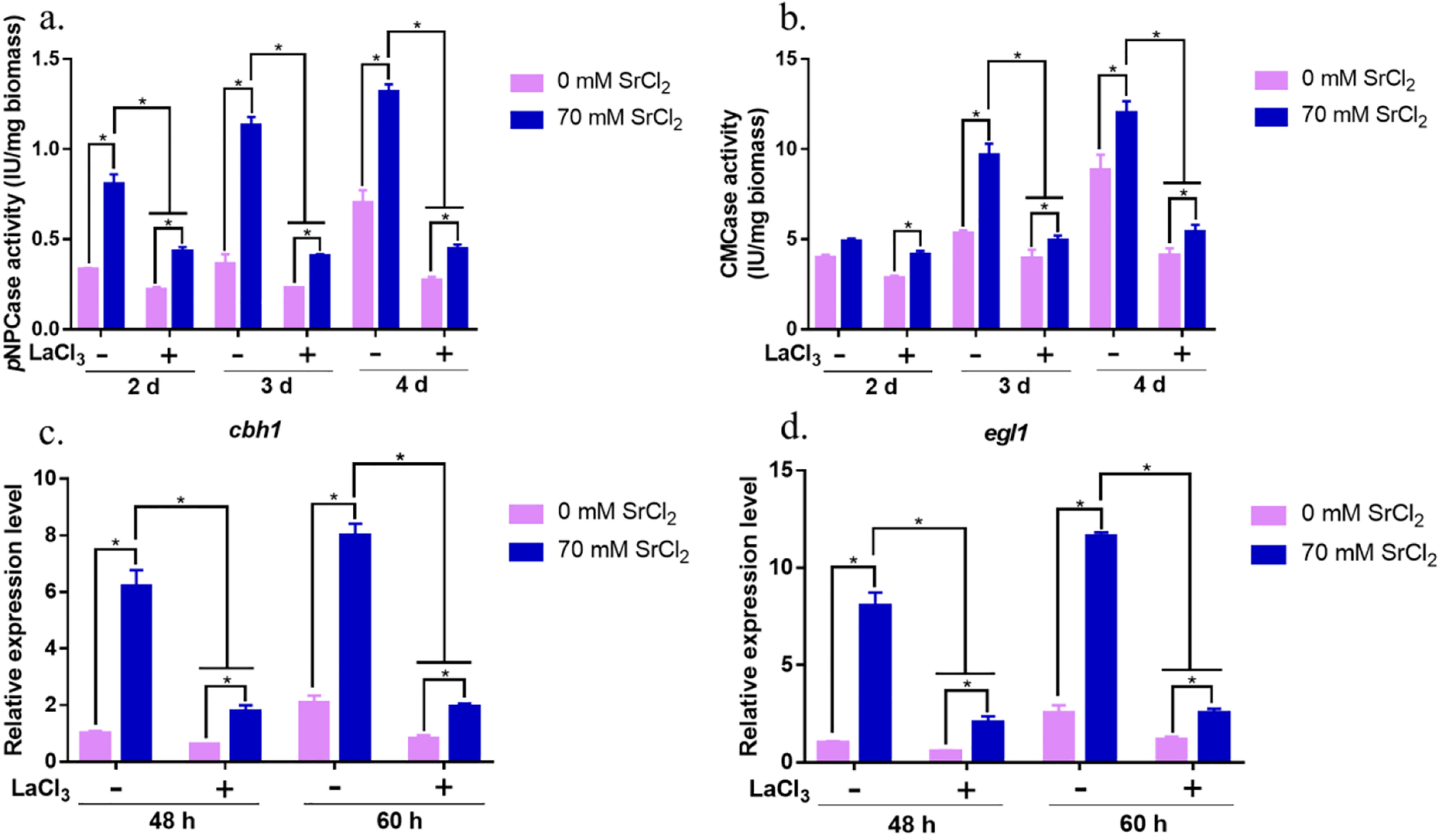
Effect of LaCl_3_ on cellulase production after Sr^2+^ treatment. *p*NPCase (**a**) and CMCase (**b**) activity were measured in the RUT-C30 strain after treatment with Sr^2+^ or LaCl_3_. The transcriptional levels of *cbh1* (**c**) and *egl1* (**d**) were determined after culturing the RUT-C30 strain in medium supplemented with 0 or 70 mM Sr^2+^ and with (+) or without (−) 5 mM LaCl_3_. The final values are represented as the mean ± standard deviation (SD) of three independent experimental results. Asterisks indicate significant differences compared to the strain without Sr^2+^ treatment (*p* < 0.05, according to Student’s *t-*test)

LaCl_3_ (5 mM) can block the Sr^2+^-induced calcium burst (Fig. 4b). To explore whether blocking the cytosolic Ca^2+^ burst attenuates Sr^2+^-induced cellulase overexpression, the activities of *p*NPCase (Fig. 5a) and CMCase (Fig. 5b) and the transcriptional levels of the vital cellulase genes *cbh1* (Fig. 5c) and *egl1* (Fig. 5d) were measured.

Under 5 mM LaCl_3_ treatment, 70 mM Sr^2+^ did not induce cellulase overproduction in *T. reesei*. The *p*NPCase and CMCase activities decreased by 1.85- to 2.97-fold and 1.17- to 2.22-fold, respectively, when LaCl_3_ was added compared to the sample without LaCl_3_ addition at 2–4 days of cultivation (Fig. 5a, b). The transcript levels of *cbh1* and *egl1* were consistent with the cellulase activity data. After LaCl_3_ addition, the expression levels of *cbh1* and *egl1* decreased to 20–30% (Fig. 5c, d). Sr^2+^-induced cellulase overexpression was significantly attenuated by LaCl_3_ treatment.

These results indicate that blocking the burst of cytosolic Ca^2+^ by LaCl_3_ substantially reduced and even eliminated the induction of cellulase production by 70 mM Sr^2+^. The cytosolic Ca^2+^ and calcium signaling pathways participate in Sr^2+^-induced cellulase overproduction in *T. reesei*. High cellulase expression induced by Sr^2+^ was due to a Ca^2+^ burst in the cytoplasm.

### Cytosolic ROS levels are significantly upregulated

**Fig. 6:**
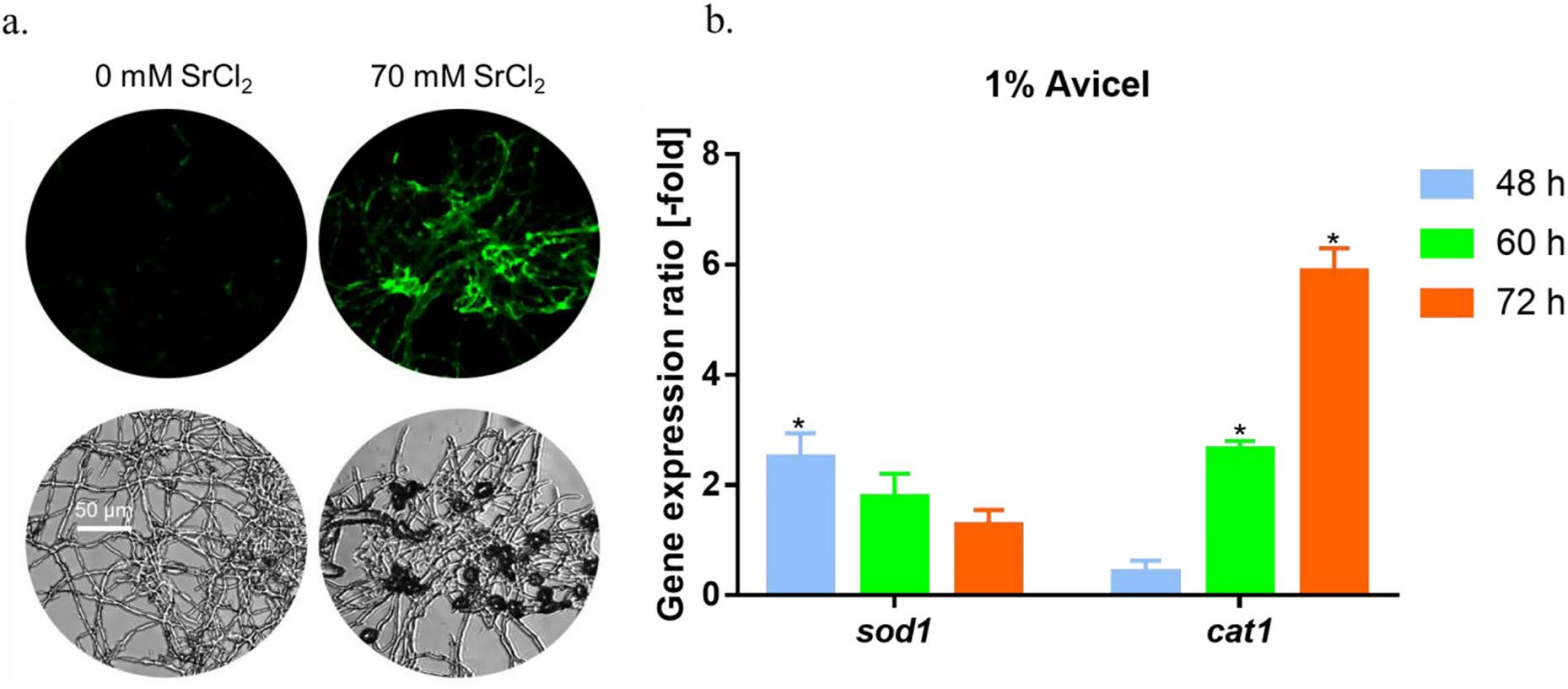
Increase in cytosolic ROS level after Sr^2+^ addition. **a** Cytosolic ROS levels were detected using the specific fluorescent probe DCFH-DA. *Trichoderma reesei* RUT-C30 strain was cultured in liquid MM with SrCl_2_ at a final concentration 0 or 70 mM for two days. To treat the hyphae, 10 μM DCFH-DA was used. The fluorescence intensity was monitored using automatic inverted fluorescence microscopy. Stronger green fluorescence indicated a higher intracellular ROS content. **b** Transcription levels of *sod1* and *cat1* were detected using RT-qPCR analysis, depicted as gene expression ratios [-fold] in RUT-C30 after treatment with 70 mM Sr^2+^ compared to RUT-C30 with no treatment on 1% Avicel for 48, 60, or 72 h. Gene expression ratios [-fold] were normalized to the corresponding gene expression at the same time point in the control (without Sr^2+^). The final values are represented as the mean ± standard deviation (SD) of three independent experimental results. Asterisks indicate significant differences, representing gene expression ratios greater than twofold or less than 0.5-fold between the treated samples and those without Sr^2+^ treatment. DIC, differential interference contrast; RT-qPCR, reverse transcription quantitative polymerase chain reaction

The ROS content in mycelia exposed to Sr^2+^ was detected using the fluorescent probe dichlorodihydrofluorescein diacetate (DCFH-DA) (Gao et al. 2018). As shown in Fig. 6a, the green fluorescence intensity of the strain exposed to 70 mM Sr^2+^ was enhanced compared to that of the control (without Sr^2+^ supplementation), indicating that Sr^2+^-induced stress increased cytosolic ROS levels. To further evaluate the influence of Sr^2+^ on the cytosolic ROS content, the transcript levels of two major antioxidant enzymes, catalase (CAT) and superoxide dismutase (SOD), were detected using RT-qPCR (Fig. 6b). After addition of 70 mM Sr^2+^, the expression of *sod1* increased by approximately 78–150% at 48–60 h. The transcript levels of *cat1* increased by 165–487% at 60–72 h, indicating that the strain was under high ROS pressure.

**Fig. 7:**
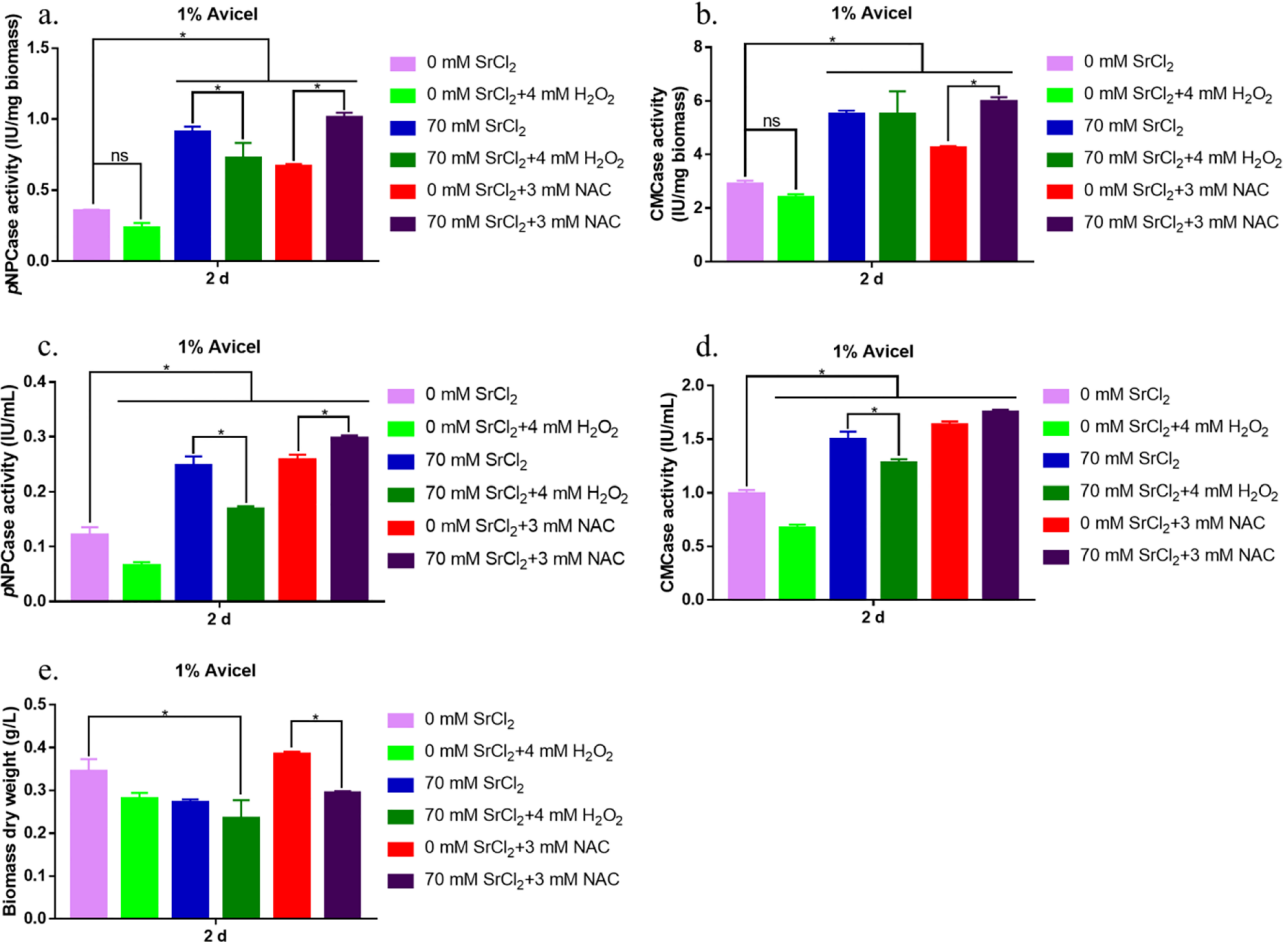
Effect of ROS on Sr^2+^-induced cellulase production. *p*NPCase activity (**a** and **c**), CMCase activity (**b** and **d**), and biomass dry weight (**e**) were determined in the RUT-C30 strain after treatment with (or without) 70 mM Sr^2+^, 4 mM H_2_O_2_, or 3 mM NAC in MM. The final values are represented as the mean ± standard deviation (SD) of three independent experimental results. Asterisks indicate significant differences compared to the strain without Sr^2+^ treatment (*p* < 0.05, according to Student’s *t-*test). MM, minimal medium; ROS, reactive oxygen species

NAC and H_2_O_2_ were used to alter the intracellular ROS content. As shown in Fig. 7a, b, adding 4 mM H_2_O_2_ reduced the levels of *p*NPCase and CMCase activity (approximately 33.1% and 16.8%, respectively). When the *T. reesei* strains were treated with 3 mM NAC, *p*NPCase and CMCase activities were markedly increased compared to those without NAC treatment (Fig. 7a–d). Under 70 mM Sr^2+^ treatment, *p*NPCase and CMCase activities also slightly increased in the presence of 3 mM NAC (approximately 11.5% and 8.4%, respectively). As shown in Fig. 7e, biomass production was measured following different chemical treatments. Addition of NAC slightly increased biomass production (approximately 11.9%), whereas treatment with H_2_O_2_ decreased biomass production (approximately 18.6%). Combined treatment with Sr^2+^ and H_2_O_2_ resulted in a large decrease in biomass (approximately 31.7%).

These results indicate that supplementation with 70 mM Sr^2+^ markedly increased ROS levels during *T. reesei* cultivation. Addition of the ROS scavenger NAC also decreased the ROS levels produced by Sr^2+^ and increased cellulase activity.

## Discussion

Metal ions are essential for primary and secondary cellular metabolic processes. Ca^2+^ (Schmoll 2011) and Mg^2+^ (Blaszczyk and Duda-Chodak 2013) are critical environmental factors that affect primary and secondary metabolism in various organisms. There are few reports on the effects of Sr^2+^, another group IIA metal ion. To the best of our knowledge, this study provides the first report that Sr^2+^ positively regulates cellulase production in *T. reesei* (Fig. 2a–e). Addition of 70 mM Sr^2+^ increased *p*NPCase and CMCase activity in the *T. reesei* wild-type strain QM6a by 53.3% and 51.6%, respectively, compared to those in the untreated control after 3 days of cultivation (Additional file 1: Figure S1a, b).

**Fig. 8:**
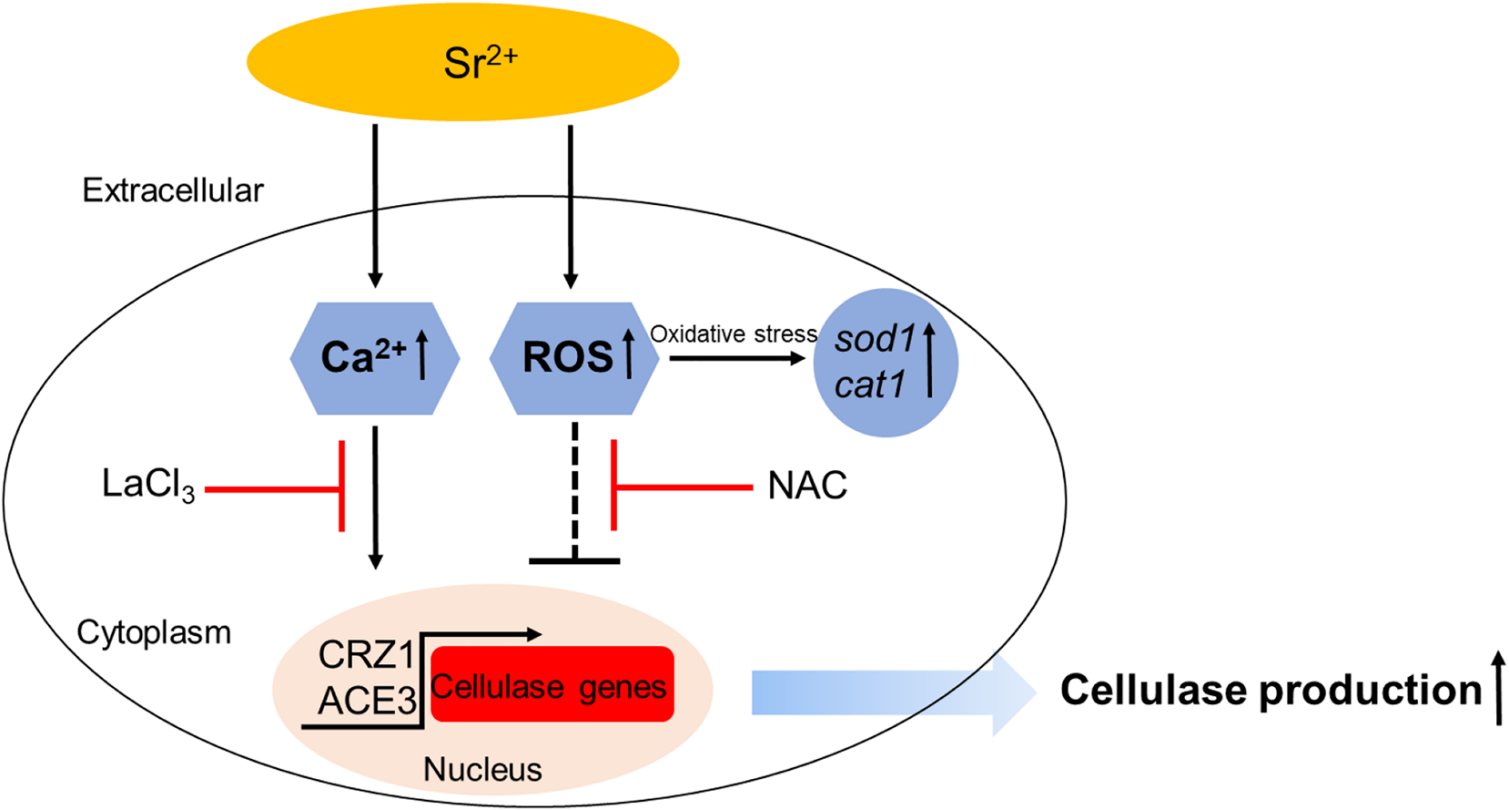
Mechanistic model of Sr^2+^-induced cellulase overexpression in *T. reesei*. Addition of 70 mM Sr^2+^ led to a significant increase in cytosolic Ca^2+^ levels, which in turn promoted overexpression of cellulase-related genes via Ca^2+^ signaling. The effect of adding NAC suggests that the cytosolic ROS burst induced by Sr^2+^ negatively affects cellulase secretion. Solid arrows indicate data supported by studies from our team, and dashed line indicate other undefined pathways. The red and black crosses represent repression. Black arrows indicate activation

Addition of 70 mM Sr^2+^ had the most significant effect on cellulase activity. The upregulated expression levels of two major cellulase genes (*cbh1* and *egl1*) and the cellulase transcription factor (*ace3*) (Fig. 3) were consistent with the cellulase activity data. Overexpression of *ace3* enables a high level of cellulase production in *T. reesei* (Luo et al. 2020). This suggests that upregulated ACE3 may mediate Sr^2+^-induced cellulase overexpression (Fig. 8). Addition of Sr^2+^ resulted in downregulation of *xyr1* expression (Fig. 3), suggesting that *xyr1* is not involved in this process.

Under Sr^2+^ pressure, treatment with Sr^2+^ led to a cytosolic Ca^2+^ burst and increased *crz1* transcription (Fig. 4c). According to previous studies, Ca^2+^ is an essential secondary messenger that can cooperate with intracellular cAMP (Benčina et al. 2005; Chen et al. 2021b), high‐osmolarity glycerol (de Castro et al. 2014) and ROS (Chen et al. 2021a; Gao et al. 2018) to regulate life activities in filamentous fungi, which is worthy of further study. However, cytosolic Ca^2+^ burst (Fig. 4a, b) and cellulase overproduction (Fig. 5a–d) were eliminated by adding the plasma membrane Ca^2+^ channel blocker LaCl_3_. These results indicate that calcium signal transduction is essential for Sr^2+^-induced cellulase production. This finding is similar to that of Chen et al. (2018), who reported that Mn^2+^ upregulates cellulase genes via calcium channels and signaling. Both Sr^2+^ and Mn^2+^ can induce cellulase overproduction; therefore, the process of Sr^2+^ induction is similar to that of the Mn^2+^/Ca^2+^ transport system in cells. In *Thermosynechococcus elongatus*, Ca^2+^/Sr^2+^ exchange affects photosystem II oxygen-evolving enzymes (Boussac et al. 2004) and electron transfer pathways from the S2 to S3 state (Boussac et al. 2015). Our results also demonstrate that Ca^2+^ plays a dominant role in Sr^2+^-induced cellulase overproduction.

According to the periodic table of elements, the number of electrons in the outermost unpaired orbital of Sr^2+^ is the same as that of Ca^2+^. This may partially explain their interaction and is worthy of further evaluated. Further studies are also required to investigate whether other metal ions interact with Ca^2+^ to regulate cellulase expression in *T. reesei*.

In this study, 70 mM Sr^2+^ inhibited growth of the strain (Fig. 1a–c), indicating that the strain was under pressure. Chen et al. (2016) reported that 100 mM Ca^2+^ repressed hyphal growth compared to that in the control. In addition, large amounts of ROS were produced after treatment with 70 mM Sr^2+^ based on analysis using an ROS probe (Fig. 6a). Gao et al. (2018) reported similar results, showing that treatment with Cu^2+^ significantly increased the intracellular ROS content in *G. lucidum*. Sr^2+^ was found to induce an increase in ROS levels, which affected cellulase production and was eliminated by adding the ROS scavenger NAC (Fig. 7a–d). In *P. brevicompactum*, treatment with high concentrations of Ca^2+^ and Mn^2+^ results in a surge in ROS levels, which regulate the synthesis of the secondary metabolite mycophenolic acid (Chen et al. 2021a, 2022). In *G. lucidum*, ROS were previously found to induce overproduction of the secondary metabolite ganoderic acid (Liu et al. 2018). These results suggest that ROS acts as a signal to regulate cellulase expression in *T. reesei*; however, this prediction requires further study. NAC can relieve ROS pressure and improve cell growth and cellulase yield, and its supplementation can improve industrial enzyme production. Large amounts of renewable lignocellulose can be hydrolyzed into sugars by cellulase to produce biofuels and chemicals. Therefore, it is crucial to decrease the price of cellulase. In industrial cellulase production, supplementation with Sr^2+^ and an ROS scavenger can increase cellulase production and improve economic efficiency.

## Conclusions

Our findings indicate that adding extracellular Sr^2+^ can rapidly and easily improve cellulase production. A putative mechanism was characterized to explain the effect of Sr^2+^ treatment on cellulase expression in *T. reesei* RUT-C30 (Fig. 8). When the strain was exposed to high concentrations of Sr^2+^ (70 mM), calcium signal transduction played a crucial role in Sr^2+^-induced cellulase production. However, the ROS content in the cytoplasm was significantly increased, thereby reducing cellulase expression. This study provides an effective approach for increasing cellulase production and insights into the effects of divalent metal ions on the life activities of filamentous fungi.

### Supplementary Information

**Additional file 1: Figure S1.** Effects of Sr^2+^ on cellulase production in the parental strain QM6a. The* p*NPCase activity (a), CMCase activity (b) of *T. reesei* QM6a were measured after culture in MM medium for 2, 3, or 4 days with (or without) 70 mM Sr^2+^. The final values are presented as the mean ± standard deviation (SD) of three independent experimental results. Asterisks indicate significant differences compared to the control (*p* < 0.05, according to Student’s *t-*test).

**Additional file 2: Table S1. **The primers used for RT-qPCR.

## Data Availability

All data generated or analyzed during this study are included and available in this published article and its additional files.

## Abbreviations

CMCase: *Endo*-β-Glucanase activity
*crz1*: Calcineurin-responsive zinc finger transcription factor 1
FPase: Filter paper activity, representing total extracellular cellulase activity
*p*NPCase: Exo-β-glucanase activity
ROS: Reactive oxygen species
RT-qPCR: Real-time quantitative PCR
