# Supplementary material for: Induction of cellulase production by Sr2+ in Trichoderma reesei via calcium signaling transduction
Source: Bioresour Bioprocess. 2022 Sep 6;9(1):96. doi: 10.1186/s40643-022-00587-3 (PMC10992071; doi:10.1186/s40643-022-00587-3)

**Figure. S1** Effects of Sr^2+^ on cellulase production in the parental strain QM6a. The *p*NPCase activity (a), CMCase activity (b) of *T. reesei* QM6a were measured after culture in MM medium for 2, 3, or 4 d with (or without) 70 mM Sr^2+^. The final values are presented as the mean ± standard deviation (SD) of three independent experimental results. Asterisks indicate significant differences compared to the control (*p*<0.05, according to Student’s *t-*test).


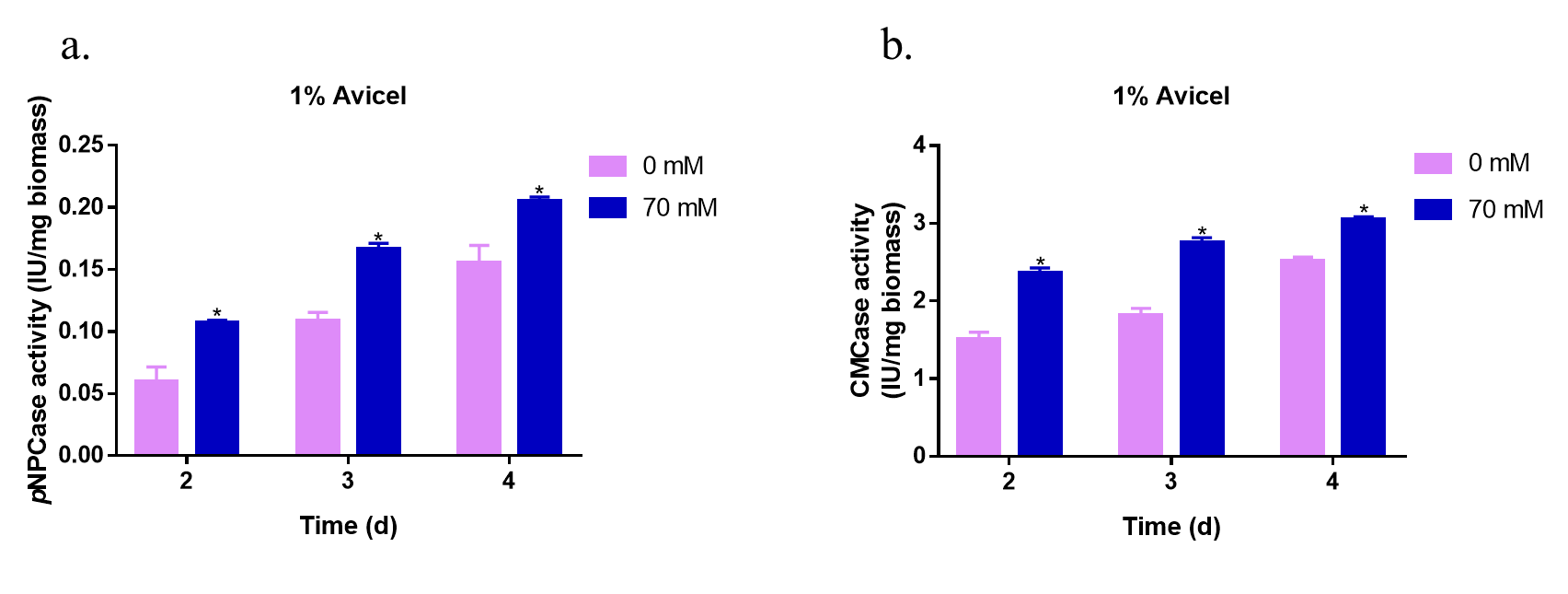

Supplement: Supplementary file 1 — Additional file 1: Figure S1. Effects of Sr2+ on cellulase production in the parental strain QM6a. The pNPCase activity (a), CMCase activity (b) of T. reesei QM6a were measured after culture in MM medium for 2, 3, or 4 days with (or without) 70 mM Sr2+. The final values are presented as the mean ± standard deviation (SD) of three independent experimental results. Asterisks indicate significant differences compared to the control (p < 0.05, according to Student’s t-test). [file 40643_2022_587_MOESM1_ESM.docx]
