# Supplementary material for: Induction of cellulase production by Sr2+ in Trichoderma reesei via calcium signaling transduction
Source: Bioresour Bioprocess. 2022 Sep 6;9(1):96. doi: 10.1186/s40643-022-00587-3 (PMC10992071; doi:10.1186/s40643-022-00587-3)
Supplement: Supplementary file 2 — Additional file 2: Table S1. The primers used for RT-qPCR. [file 40643_2022_587_MOESM2_ESM.docx]

**Table. S1** The primers used for RT-qPCR

| **Primer** | **oligos Sequences (5’ to 3’)** |
| --- | --- |
| Q*sar1*-1 | TGGATCGTCAACTGGTTCTACGA |
| Q*sar1*-2 | GCATGTGTAGCAACGTGGTCTTT |
| Q*cbh1*-1 | CTCCATCTCCGAGGCTCTTACC |
| Q*cbh1*-2 | GCAAGTGCCGCCATATCTGTTAT |
| Q*egl1*-1 | GCAGCCTCACCATGAACCAGTA |
| Q*egl1*-2 | CACCGTCAGAGTCCAGGAGATAC |
| Q*ace3*-1 | GCCAAGTGCGAGTACCTCAG |
| Q*ace3*-2 | GCTGGTCGCTCTTCTTCCTC |
| Q*xyr1*-1 | CTTCCTCCTCCTGCTCATCG |
| Q*xyr1*-2 | TCGTGTGCCCTAACAATGGTC |
| Q*sod1*-1 | CCTCTGCCGGCCCTCACTTC |
| Q*sod1*-2 | AATGGTGCCCTTGGCGTTGC |
| Q*cat1*-1 | GTGCGGCAGAGACCCGGATT |
| Q*cat1*-1 | TCGGGATCTGCCTGGTCGGT |
| Q*crz1*-1 | CCAGCAGATGCCGGACACCA |
| Q*crz1*-2 | GTGCATATCGCCGCCCATGC |
|  |  |
